# T Regulatory Cells Control Susceptibility to Invasive Pneumococcal Pneumonia in Mice

**DOI:** 10.1371/journal.ppat.1002660

**Published:** 2012-04-19

**Authors:** Daniel R. Neill, Vitor E. Fernandes, Laura Wisby, Andrew R. Haynes, Daniela M. Ferreira, Ameera Laher, Natalie Strickland, Stephen B. Gordon, Paul Denny, Aras Kadioglu, Peter W. Andrew

**Affiliations:** 1 Department of Infection, Immunity and Inflammation, University of Leicester, Leicester, United Kingdom; 2 MRC Harwell, Mammalian Genetics Unit, Harwell Science and Innovation Campus, Oxfordshire, United Kingdom; 3 Liverpool School of Tropical Medicine, Pembroke Place, Liverpool, United Kingdom; Harvard Medical School, United States of America

## Abstract

Streptococcus pneumoniae is an important human pathogen responsible for a spectrum of diseases including pneumonia. Immunological and pro-inflammatory processes induced in the lung during pneumococcal infection are well documented, but little is known about the role played by immunoregulatory cells and cytokines in the control of such responses. We demonstrate considerable differences in the immunomodulatory cytokine transforming growth factor (TGF)-β between the pneumococcal pneumonia resistant BALB/c and susceptible CBA/Ca mouse strains. Immunohistochemistry and flow cytometry reveal higher levels of TGF-β protein in BALB/c lungs during pneumococcal pneumonia that correlates with a rapid rise in lung Foxp3^+^Helios^+^ T regulatory cells. These cells have protective functions during pneumococcal pneumonia, because blocking their induction with an inhibitor of TGF-β impairs BALB/c resistance to infection and aids bacterial dissemination from lungs. Conversely, adoptive transfer of T regulatory cells to CBA/Ca mice, prior to infection, prolongs survival and decreases bacterial dissemination from lungs to blood. Importantly, strong T regulatory cell responses also correlate with disease-resistance in outbred MF1 mice, confirming the importance of immunoregulatory cells in controlling protective responses to the pneumococcus. This study provides exciting new evidence for the importance of immunomodulation during pulmonary pneumococcal infection and suggests that TGF-β signalling is a potential target for immunotherapy or drug design.

## Author Summary

*Streptococcus pneumoniae* is a major human bacterial pathogen that causes a wide range of diseases including pneumonia, meningitis, sepsis and ear infections. The bacterium is responsible for around 1.2 million deaths per year, mostly in high-risk groups such as children, the elderly and those with a weakened immune system. Infection with the pneumococcus can induce a wide-variety of immune responses and disease symptoms and it is not known why some people are more resistant to infection than others. Here, we identify an important role in natural resistance against pneumococcal pneumonia for a group of cells – known as T regulatory cells – that control the immune response to pneumococcal infection. In mice, strong T regulatory cell responses correlate with resistance to invasive pneumococcal pneumonia. Disease-resistance can be boosted by administering T regulatory cells to highly susceptible mice or inhibited by blocking the activity of these cells in resistant mice. These results advance our understanding of the host immunity differences that underpin resistance to pneumococcal pneumonia and offer hope that in the future we might boost resistance in susceptible individuals through modulation of their immune system.

## Introduction

*Streptococcus pneumoniae* (the pneumococcus) is an important human pathogen that accounts for significant morbidity and mortality, particularly in high-risk groups such as children, the elderly and the immunocompromised. Determination of host protective immune responses to pneumococcal infection is highly desirable, not least because an improved understanding of the cellular and molecular interactions of host and pathogen might enable the design of specific, targeted therapies that are able to overcome the limitations of current treatment and prevention strategies.

It has long been thought that neutrophils are the primary mediators of early defence against pulmonary pneumococcal infection [1]–[3] but more recently a key role for T cells in the earliest stages of immunity against the pneumococcus has emerged. It has been noted that T cells infiltrate the lungs of intranasally-infected mice at an early time post-infection and that the peak of infiltration coincides with a cessation in pneumococcal growth in the lungs [4]. Furthermore, it has been demonstrated that CD4^+^ T cells contribute to protective immunity to invasive pneumococcal pneumonia, as MHC-II-deficient mice, which have severely reduced numbers of CD4^+^ T cells, are highly susceptible to infection [5]. However, whilst there is an expanding body of work outlining the roles of inflammatory T cell subsets [6]–[9], little has been made of regulatory and anti-inflammatory T cells and their influence on the outcome of pneumococcal pneumonia. This is an important gap in our understanding of the immunology of pneumococcal disease.

Several immune cell types, including tolerogenic DCs [10], myeloid suppressor cells [11], IL-10-producing CD4^+^ T cells [12], B cells [13] and Foxp3^+^ T regulatory cells [14], have confirmed roles in the modulation and inhibition of inflammation in the context of infection. Of these immunomodulatory cells, Foxp3^+^ T regulatory cells are probably the best characterized. These cells have emerged as essential components of the mammalian immune system and play crucial roles in immune homeostasis as well as limiting infection-associated inflammation and facilitating resolution of tissue damage post-infection [15], [16]. Indeed, for West Nile Virus, a link has been made between T regulatory cell activity and symptomatic versus asymptomatic infection [17].

Little is known about the actions of immunomodulatory cytokines and T regulatory cells during infection with *S. pneumoniae*, but recent data suggest an importance of immunomodulation in anti-pneumococcal responses. Intraperitoneal administration of *S. pneumoniae* serotype 1 capsular polysaccharide induces CD8^+^CD28^−^ T-cells with a regulatory phenotype [18] that synthesize both IL-10 and TGF-β and are immunosuppressive for CD4^+^ T cells *in vivo* and *in vitro*. However, whether regulatory cells play protective roles during infection with invasive *S. pneumoniae* strains remained to be elucidated. Although it has previously been observed that CD25^+^ T cells are induced during pneumococcal pneumonia [5] and that the administration of heat-killed *S. pneumoniae* to mice can suppress allergic airway disease by the induction of T regulatory cell expansion [19], the studies reported in this paper now provide new evidence for the important protective role performed by T regulatory cells in an *in vivo* model of invasive pneumococcal pneumonia.

The need to address the role of immunomodulatory cells and cytokines in pneumococcal infection is pressing, not least because it is unclear at present whether their actions are protective or detrimental to the outcome of infection. For example, IL-10 is a cytokine produced by T regulatory cells that plays key protective roles in limiting inflammation in a number of diseases through its pleiotropic inhibitory effects on immune cells, but it has also been shown to impair defense mechanisms in pneumococcal pneumonia (albeit post influenza infection) [20]. In a recent study, adenoidal cells from children testing positive for pneumococcal carriage were found to contain higher numbers of Foxp3^+^ T regulatory cells than those taken from children without pneumococcal carriage suggesting an immunosuppressive role for these cells. Furthermore, *in vitro*, pneumococcal whole cell antigen induced T regulatory cell proliferation and production of IL-10 [21]. In this paper, we have now addressed the role of T regulatory cells *in vivo* during invasive pneumococcal pneumonia.

To determine the involvement of T regulatory cells and immunomodulatory cytokines in resistance to pneumococcal infection, we utilized two mouse strains that have strikingly different susceptibility to pneumococcal pneumonia [22], [23]. BALB/c mice are highly resistant to respiratory challenge against a wide range of invasive pneumococci and confine their infection to the lung (without developing sepsis) and then eliminate it within 7 days. By contrast, CBA/Ca mice succumb quickly to respiratory infection, developing septicaemia and dying within 24 hours of infection [22].

Linkage mapping of the offspring of BALB/c and CBA/Ca intercrosses revealed a major locus on mouse chromosome 7, the *Spir1* (*Streptococcus pneumoniae* infection resistance 1) locus, that influences survival after infection with *S. pneumoniae* [23]. The *Tgfb1* gene is located within this region of chromosome 7 and this prompted us to investigate the contribution of TGF-β and its downstream targets – including T regulatory cells - to the differing susceptibilities of BALB/c and CBA/Ca mice to invasive pneumococcal pneumonia. These studies have unearthed an important but hitherto unappreciated role for T regulatory cells in limiting immune-mediated damage in the lungs during pneumococcal pneumonia. This is, to our knowledge, the first study identifying a protective role for T regulatory cells during invasive pneumonia and it is to be hoped that the findings herein may facilitate the design of pneumococcal therapies aiming to improve T regulatory cell responses in susceptible or infected individuals.

## Results

### TGF-β signaling is regulated differentially in BALB/c and CBA/Ca mice during pneumococcal pneumonia

**Figure 1:**
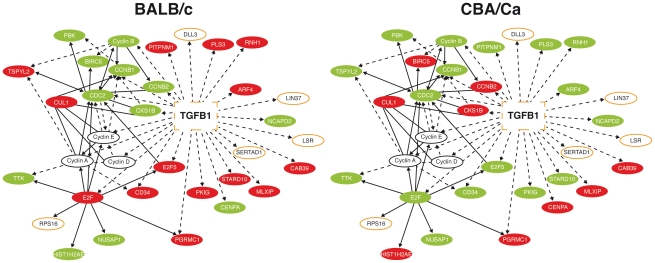
Many *Tgfβ1* interaction partners show opposing expression profiles in BALB/c and CBA/Ca mice at 6 hours p.i. Ingenuity pathway analysis of microarray data from 6 hours p.i. Red=Gene significantly upregulated as compared to t=0 levels. Green=Down-regulated. BALB/c levels are compared to BALB/c t=0 expression and CBA/Ca levels are compared to CBA/Ca t=0 expression. Orange outline=Gene found within *Spir1* susceptibility-determining locus. Solid lines indicate direct interactions, dotted lines indicate indirect interactions.

In a preliminary study, microarray comparison of gene expression in lung tissue from BALB/c or CBA/Ca mice following intranasal infection with *S. pneumoniae* indicated significant differential regulation of the TGF-β signalling pathway between the two strains (Figure 1). At 6 hours post-infection (p.i.) many of the interaction partners of *Tgfb1* showed opposing regulation in BALB/c and CBA/Ca mice.

**Figure 2:**
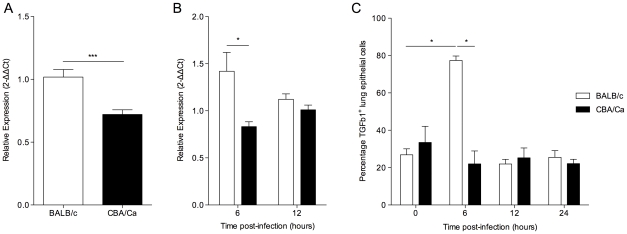
*tgfb1* expression and TGF-β production is upregulated in BALB/c lungs in contrast to CBA/Ca lungs during pneumococcal infection. Relative gene expression of *tgfβ1* in lungs of BALB/c and CBA/Ca mice before (A) and during (B) pneumococcal infection. Data were normalised against a housekeeping gene (Hprt1) and are presented using BALB/c results as a calibrator (A) or else using BALB/c or CBA/Ca sham-infection data from the appropriate time-point as calibrators (B). For 6 and 12 hours p.i. data for each strain was calibrated against sham-infection data for that strain at the same time-point. i.e. BALB/c to BALB/c sham and CBA/Ca to CBA/Ca sham. (C) Antibody staining of TGF-β in airway epithelial cells from BALB/c and CBA/Ca mice during pneumococcal infection. *=p<0.05, ***=p<0.005. For all figures data represent mean +/− SEM. Data are representative of two independent experiments with 5 mice per time-point per strain and per treatment group.

As TGF-β and many of its interaction partners are found within the *Spir1* susceptibility locus (Figure 1) [23], *Tgfb1* was selected for further analysis. qRT-PCR analysis was performed on lung tissue from intranasally-infected BALB/c and CBA/Ca mice to determine *Tgfb1* expression over the course of pneumococcal pneumonia. Strikingly, expression of *Tgfb1* was significantly higher in BALB/c than in CBA/Ca mice both before infection and at 6 hours p.i. (Figure 2 A and B).

To investigate whether the observed differences in *Tgfb1* gene expression were reflected in changes in TGF-β1 protein in the lungs, immunohistochemical analysis was performed on lung sections (Figure 2 C). A significant increase in the percentage of epithelial cells staining TGF-β1^+^ in the lungs of BALB/c mice, but not CBA/Ca mice was observed between 0 and 6 hours p.i. Interestingly, the percentage of TGF-β1^+^ epithelial cells in BALB/c lungs returned to pre-infection levels by 12 hours p.i., perhaps as the result of cleavage of active TGF-β protein from the surface of the cells. These data indicate a rapid and robust increase in TGF-β1 synthesis and export by BALB/c airway epithelial cells in response to infection, which is absent in CBA/Ca mice.

### BALB/c mice display a greater expansion and recruitment of Foxp3^+^ cells to the lungs than do CBA/Ca mice during pneumococcal pneumonia

**Figure 3:**
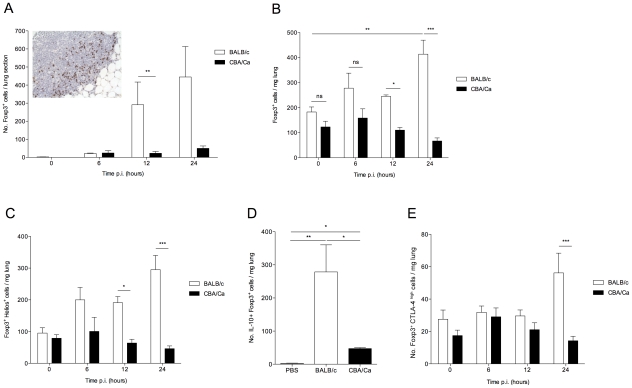
IL-10-producing natural T regulatory cells are rapidly recruited to the lung in *S. pneumoniae*-infected BALB/c mice. All results are from intranasal-infection of mice with wild-type *S. pneumoniae* D39. (A) Foxp3 immunostaining of lung sections taken from BALB/c and CBA/Ca mice during pneumococcal infection. Inset shows example staining from 12 hour p.i. BALB/c mouse. Foxp3^+^ cells stain brown. (B) Number of Foxp3^+^ T regulatory cells, (C) Number of Foxp3^+^Helios^+^ cells, (D) number of Foxp3^+^IL-10^+^ cells and (E) number of Foxp3^+^CTLA-4^+^ cells per mg lung in *S. pneumoniae*-infected BALB/c and CBA/Ca mice, as identified by flow cytometry. Data in (D) are from 24 hrs p.i., PBS group contains both BALB/c and CBA/Ca mice. White bars=BALB/c, black bars=CBA/Ca. *'s indicate significant difference, where *=p<0.05, **=p<0.01, and ***=p<0.005. For all graphs data represent mean +/− SEM. All results are representative of between 2–4 independent experiments with >4 mice per group.

T regulatory cells are both a source and a target of TGF-β, so the numbers and function of these cells were investigated in the lungs of BALB/c and CBA/Ca mice before and during pneumococcal infection. Staining of lung tissue sections for Foxp3 revealed no differences in the numbers of Foxp3^+^ cells in the lungs of uninfected CBA/Ca and BALB/c mice but substantially increased numbers in BALB/c at both 12 and 24 hours p.i. (p<0.05), with no significant changes apparent in the CBA/Ca strain (Figure 3 A). The correlation between T regulatory cell numbers and the effectiveness of the ongoing immune response in the lung is readily apparent as bacterial CFUs in the lungs of BALB/c mice fall rapidly by 24 hours p.i., while bacterial numbers in CBA/Ca lungs increase over the same time period despite similar CFUs for both mouse strains at 12 p.i. (Figure S1 A) [22].

To further characterize the Foxp3^+^ cells identified in the lungs during pneumococcal infection, flow cytometry was performed on lung cells from infected mice. Intracellular staining for Foxp3 confirmed the immunohistochemistry finding that whilst there was no apparent difference in the number of T regulatory cells in the lungs of uninfected BALB/c and CBA/Ca mice (Figure 3 B), by 24 hours p.i., T regulatory cell numbers had risen approximately 2.5-fold in the lungs of BALB/c mice (p<0.01) but remained unchanged in CBA/Ca mice (p>0.05), as compared to uninfected levels (Figure 3 B). This increase in T regulatory cell numbers also correlate with significantly reduced CFUs in lungs of BALB/c mice compared to CBA/Ca mice at equivalent time points (Figure S1 A).

Regulatory T cells can develop in the thymus or from CD4^+^ T cells in the periphery. The thymus-derived cells are commonly referred to as natural T regulatory cells (nTregs) and it has been suggested that they can be distinguished from the cells developing in the periphery (induced T regulatory cells; iTregs) by their expression of the Ikaros family transcription factor, Helios [24]. However, more recently it has been observed that Helios is expressed on activated T cells of all subsets and so is perhaps not suitable as an nTreg marker [25]. Thus, in this study we have used Helios as a marker for activated T regulatory cells. Analysis of Helios expression in lung cells during our experiments revealed that the increase in T regulatory cells in BALB/c lungs during pneumococcal pneumonia was due to increased Helios^+^ cells (Figure 3 B and C). Foxp3^+^Helios^+^ cells increased approximately 3-fold over the first 24 hours p.i. in BALB/c mice, but not in CBA/Ca mice (p<0.001), indicating that BALB/c T regulatory cells are activated rapidly following infection. By contrast, there was no significant increase in Foxp3^+^Helios^negative^ cells in the lungs of either BALB/c or CBA/Ca mice over the course of the infection (data not shown). The increase in T regulatory cells in BALB/c lungs following infection is rapid, supporting a greater role for nTregs than for iTregs, which can take several days to differentiate in the periphery in response to TGF-β and low doses of antigen.

To assess whether lung T regulatory cells actively secrete immunomodulatory cytokines during pneumococcal infection, the number of IL-10^+^ T regulatory cells was assessed (Figure 3 D). T regulatory cell-derived IL-10 has an important role in determining resistance or susceptibility to infection against a range of pathogens [26]–[27] and has been shown to influence susceptibility to disease in pneumococcal pneumonia [28]. In our study, a significant increase in IL-10^+^ T regulatory cells was readily apparent in both BALB/c (p<0.01) and CBA/Ca (p<0.05) mice compared to PBS controls at 24 hours p.i. However, the number of IL-10^+^ T regulatory cells in BALB/c mice was significantly higher than in infected CBA/Ca mice (p<0.05).

In addition to IL-10, several other markers – including PD-L1 [29] and CTLA-4 [30] - have been suggested to play a role in T regulatory cell immunosuppressive functions. We assessed expression of PD-L1 and CTLA-4 by T regulatory cells in the lungs of BALB/c and CBA/Ca mice. We observed barely detectable expression of PD-L1 on T regulatory cells from either BALB/c or CBA/Ca mice (data not shown) and low-level expression of CTLA-4 on the majority of T regulatory cells from both strains (data not shown). However, we also observed a population of CTLA-4^high^ T regulatory cells in both strains of mice (Figure 3 E). There was a significant difference in CTLA-4^high^ T regulatory cell numbers between BALB/c and CBA/Ca mice at 24 hours p.i. (p<0.001), with BALB/c lungs containing substantially more CTLA4^high^ cells (Figure 3 E). This increase in CTLA-4^high^ T regulatory cells in BALB/c mice could also be observed in the spleen at 24 hours p.i. (Figure S2), suggesting the presence of a circulating population of highly suppressive T cells. This result further highlights the strong regulatory environment generated in BALB/c mice following infection.

To rule out the possibility that the differences in TGF-β signaling between BALB/c and CBA/Ca mice were affecting disease-resistance through modulation of T_H_17 responses, the production of IL-17 was assessed in the lungs of BALB/c and CBA/Ca mice at 24 hours p.i. (Figure S3). We observed only a small population of IL-17^+^ cells in either mouse strain and no significant difference in CD4^+^IL-17^+^ cells was observed.

### CBA/Ca mice show increased interferon gamma and increased cellular apoptosis during pneumococcal pneumonia

**Figure 4:**
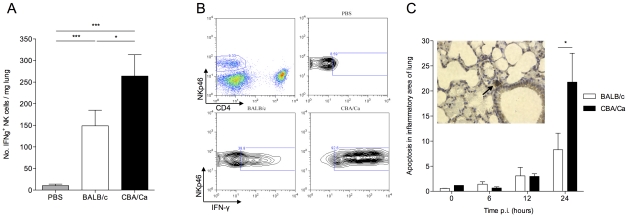
CBA/Ca mice display uncontrolled lung inflammation and associated apoptosis following *S. pneumoniae* infection. All results are from intranasal-infection of mice with wild-type *S. pneumoniae* D39. (A) Number of IFNγ^+^NKp46^+^CD4^−^ cells per mg lung in PBS-treated or *S. pneumoniae*-infected BALB/c and CBA/Ca mice at 24 hours p.i. (B) Top left shows gating of NK cells in lung homogenate. Remaining panels show example IFNγ-staining from PBS-treated CBA/Ca or *S. pneumoniae*-infected BALB/c and CBA/Ca mice at 24 hours p.i. (C) Proportion (%) of apoptotic cells in areas of inflammation within lungs of *S. pneumoniae*-infected BALB/c and CBA/Ca mice. Inset shows apoptotic cell (arrow) within area of inflammation as defined by presence of inflammatory infiltrate. White bars=BALB/c, black bars=CBA/Ca. *'s indicate significant difference, where *=p<0.05 and ***=p<0.005. For all graphs data represent mean +/− SEM. Results in (A and B) are representative of 3 independent experiments with >4 mice per group. Results in (C) are from a single experiment with 5 mice per group. PBS-treated groups contained both BALB/c and CBA/Ca mice.

The strong immunomodulatory environment generated in BALB/c lungs during pneumococcal pneumonia accounts for the lower levels of pro-inflammatory cytokine seen in these mice p.i. (Figure 4). Production of interferon (IFN)-γ in CBA/Ca lungs at 24 hours p.i. was significantly higher than in BALB/c lungs (p<0.05) (Figure 4 A), and over 90% of NKp46^+^ NK cells were found to be IFN-γ^+^, as compared to 30–50% in BALB/c mice (Figure 4 B). NK cells accounted for over 80% of IFN-γ-producing cells in both BALB/c and CBA/Ca mice, with the remaining 20% coming from an unidentified CD45^+^CD4^−^CD8^−^NKp46^−^ cell type (data not shown). The higher levels of IFN-γ production in CBA/Ca mice suggests that mechanisms limiting pro-inflammatory cytokine production are more effective in BALB/c than in CBA/Ca lungs during pneumococcal pneumonia. IFN-γ is known to induce apoptosis in a range of cell types including lung epithelial cells [31], [32] and so it is possible that modulation of IFN-γ production is one mechanism by which T regulatory cells may help maintain epithelial barrier integrity and confine the bacterial infection to the lung.

Intriguingly, the immunomodulation occurring in BALB/c lungs at early time-points following infection appears to be a specific process rather than a general dampening of immune responses as BALB/c mice generate strong neutrophil responses in the lung following intra-nasal infection (Figure S4 A) that are thought to be critical in bacterial clearance [22]. Little influx of neutrophils is observed in CBA/Ca mice over the first 24 hours p.i. and so this response must be absent or else occurring after bacteria have already disseminated to the blood stream (Figure S1 and Figure S4 A).

To determine whether reduced T regulatory cell numbers and increased pro-inflammatory cytokine production correlated with tissue damage in CBA/Ca mice, apoptosis was assessed in the inflammatory loci within the lungs of *S. pneumoniae*-infected mice (Figure 4 C). At 24 hours p.i., a time point at which BALB/c lungs contain more T regulatory cells and less IFN-γ than CBA/Ca mice (Figure 3 A, B and Figure 4 A), a significantly higher proportion of cells in the inflamed tissue had undergone apoptosis in the lungs of CBA/Ca than in BALB/c mice (p<0.05) (Figure 4 C). At the same time p.i. (and 12 hrs previously), bacterial dissemination from the lungs into the bloodstream had already occurred in CBA/Ca mice, but not in BALB/c mice, which had no septicaemia (Figure S1 B).

### Inhibition of TGF-β1 impairs BALB/c resistance to pneumococcal pneumonia

**Figure 5:**
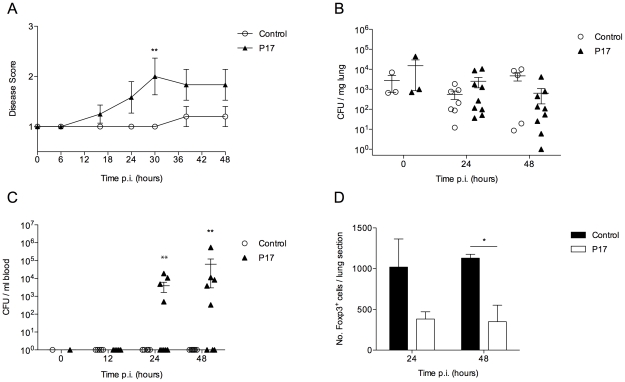
Inhibition of TGF-β activity impairs BALB/c resistance to pneumococcal infection. All results are from intranasal-infection of mice with wild-type *S. pneumoniae* D39. (A) Mean animal disease score in *S. pneumoniae*-infected PBS-treated or P17-treated BALB/c mice over a 48 hour time-course. Normal mice score 1. (B) Number of bacteria per mg lung tissue and (C) per ml blood in P17- (▴) or PBS-treated (○) mice. CFU=colony forming units. (D) Quantification of Foxp3^+^ immunostaining in antibody-stained lung sections from P17- (black bars) or PBS-treated (white bars) *S. pneumoniae*-infected mice. *'s indicate significant difference, where *=p<0.05 and **=p<0.01. For all graphs data represent mean +/− SEM. Results in (A–D) are representative of 2 independent experiments with >5 mice per group.

Having demonstrated differences in TGF-β production and T regulatory cell activity over the course of infection in BALB/c and CBA/Ca mice, we next investigated the potential of TGF-β1-inhibition in BALB/c mice to influence their resistant phenotype to pneumococcal pneumonia. If TGF-β had a significant role to play in host defence against pneumonia, then inhibition of it would render the BALB/c host more susceptible. A short synthetic peptide (P17) was utilized to inhibit TGF-β activity in BALB/c animals. The ability of P17 to effectively inhibit TGF-β1 and TGF-β2 activity is well documented [33], [34]. P17, or PBS as a control, was administered to BALB/c mice 1 hour prior to intranasal challenge with *S. pneumoniae* and then again at 6 hours p.i. Strikingly, mice treated with the P17 peptide developed visible signs of disease earlier than PBS-treated control animals and by 48 hours p.i. all ten P17-treated animals, but only one mouse from the control group, displayed visible signs of disease (Figure 5 A). Furthermore, 50% of P17-treated mice had to be culled due to illness, while 100% of the control group survived (data not shown). Although mice from both treatment groups showed similar lung CFUs by 24 and 48 hours p.i., (Figure 5 B), P17-treated BALB/c mice had significantly higher CFUs in their blood (Figure 5 C). Whilst none of the PBS-treated BALB/c mice developed bacteraemia over the 48-hour experiment, 50% of the P17-treated animals had substantial levels of bacteria in their blood by 48 hours p.i., indicating that inhibition of TGF-β promotes bacterial seeding from lungs to blood. This is a key finding as BALB/c mice intranasally infected with *S. pneumoniae* do not develop septicaemia [22]. Therefore, in the absence of an effective T regulatory cell and TGF-β response, resistant mice become susceptible to pneumococcal pneumonia, developing sepsis (which control mice do not) and substantially increased mortality.

As TGF-β is thought to be required for T regulatory cell development, we investigated whether P17 treatment had had any effect on T regulatory cell numbers in the lungs of infected BALB/c mice. Analysis of Foxp3-stained lung sections from *S. pneumoniae*-infected PBS-treated and P17-treated mice confirmed that P17 treatment substantially reduced numbers of Foxp3^+^ cells in the lungs of infected BALB/c mice at both 24 and 48 hours p.i., which correlated with development of sepsis in these mice (Figure 5 D).

### Adoptive transfer of T regulatory cells prolongs survival of susceptible CBA/Ca mice

**Figure 6:**
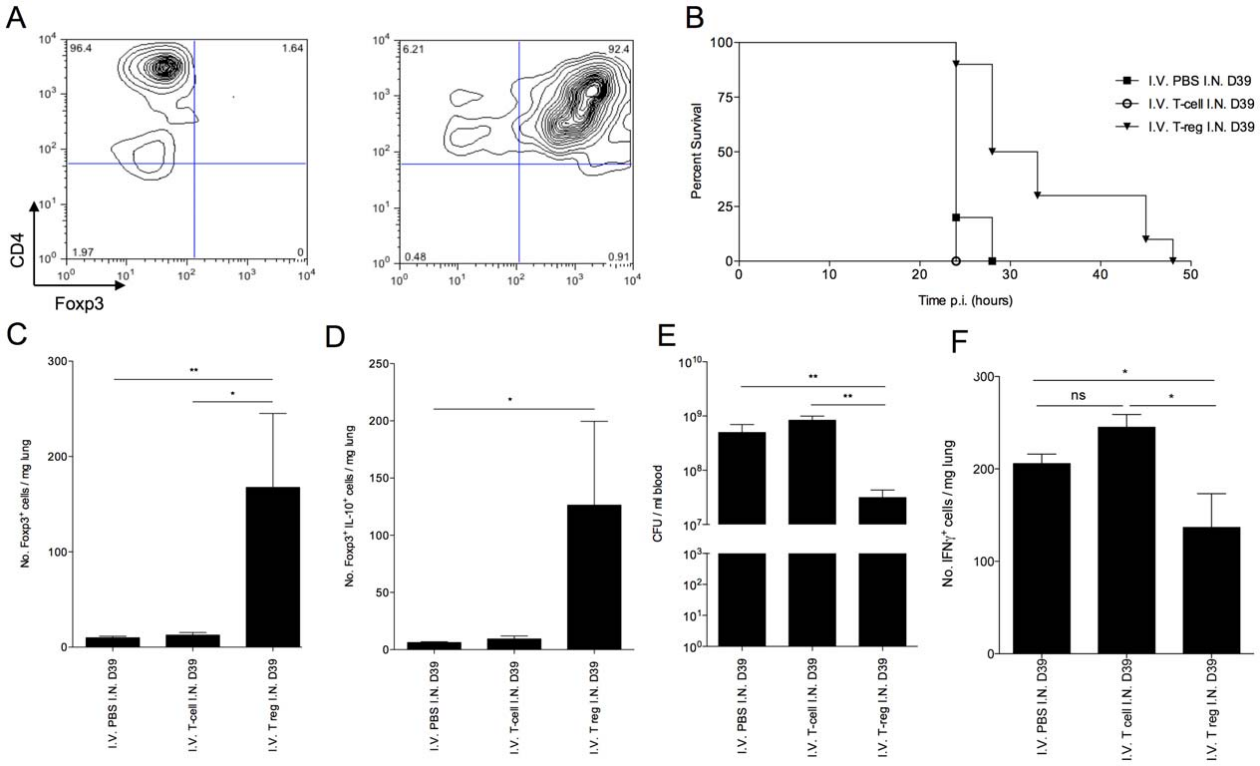
Adoptive transfer of *in vitro* generated Foxp3^+^ T regulatory cells improves CBA/Ca survival in pneumococcal infection. (A) CD4 and Foxp3 FACS staining of isolated CBA/Ca CD4^+^, CD25^−^ splenocytes before (left column) and after (right column) 5 days culture with anti-CD3, anti-CD28 and TGF-β. 5 day cultured cells were used for adoptive transfer. All results in (B–D) are from intranasal-infection of mice with wild-type D39 *S. pneumoniae*. (B) Survival of *S. pneumoniae*-infected CBA/Ca mice that had received PBS (▪), CD4^+^ T cells (○), or T regulatory cells (▾) i.v. prior to intranasal infection with *S. pneumoniae*. Survival of T regulatory cell treatment group is significantly different to both CD4^+^ T cell treatment group (p<0.01) and PBS treatment group (p<0.01) (C) Number of Foxp3^+^ cells and (D) number of IL-10^+^Foxp3^+^ cells per mg lung at 24 hours p.i. (E) Number of bacteria/ml blood at 24 hour post-infection. (F) Number of IFN-γ^+^ cells per mg lung at 24 hours p.i.. CFU=colony forming units. I.N.=Intranasal. I.V.=Intravenous. *'s indicate significant difference, where *=p<0.05 and ***=p<0.001. For all graphs data represent mean +/− SEM. Results in (A) are representative of 5 independent experiments. Results in (B–F) are representative of two independent experiments with >6 mice per group.

We next investigated whether increased numbers of T regulatory cells in CBA/Ca mice might boost their resistance to pneumococcal pneumonia. To generate sufficient numbers of cells for adoptive transfer experiments, we differentiated inducible T regulatory cells from CD4^+^, CD25^negative^ T cells *in vitro*, by culture with TGF-β in the presence of anti-CD3 and anti-CD28 stimulation. Whilst these cells have differentiation requirements more similar to iTregs than nTregs, they share similar immunosuppressive qualities with nTregs, such as IL-10 production and CTLA-4 expression [35]. The differentiation protocol yielded approximately 90% CD4^+^Foxp3^+^ T regulatory cells, with a few contaminating CD4^+^Foxp3^negative^ T cells (Figure 6 A).

Adoptive transfer of *in vitro* generated CBA/Ca Foxp3^+^ T regulatory cells, via the tail vein, 2 hours prior to intranasal infection with *S. pneumoniae* led to a significant increase in mean survival of CBA/Ca mice (p<0.01) (Figure 6 B). By contrast, CD4^+^Foxp3^negative^ T cells generated in the same culture conditions, but without TGF-β, and transferred into a separate group of mice to control for contaminating T cells in the T regulatory cell population, failed to increase survival (Figure 6 B). At 24 hours p.i., a significant increase (p<0.01) in total T regulatory cells (Figure 6 C) and IL-10^+^ T regulatory cells (Figure 6 D) was observable in the lungs of mice that had received i.v. administration of T regulatory cells, in contrast to mice that had received PBS or CD4^+^Foxp3^negative^ T cells i.v. This finding clearly demonstrated an ability of the *in vitro* generated T regulatory cells to influence immune responses, leading to increased host survival, following transfer into the tail vein. Crucially, the bacterial load in blood at 24 hours p.i. was significantly lower in mice that had received T regulatory cells than either of the other two treatment groups (p<0.001) (Figure 6 E). This suggests reduced translocation of pneumococci from lungs to blood during pneumonia as a result of T regulatory cell activity. This is a significant result as the CBA/Ca strain is highly susceptible to invasive pneumococcal pneumonia, and yet transfer of T regulatory cells alone was sufficient to double survival time. Furthermore, the increased survival and decreased seeding of bacteria to blood in CBA/Ca mice that had received T regulatory cells correlated with a decrease in the number of IFN-γ^+^ cells in the lungs, suggesting an improved generation of an immunomodulatory environment in these mice (Figure 6 F). The decreased levels of pro-apoptotic IFN-γ in these mice may contribute to the decreased seeding of bacteria from lungs to blood (Figure 6 E).

### Resistance to invasive pneumococcal pneumonia in outbred mice correlates with strong T regulatory cell responses

**Figure 7:**
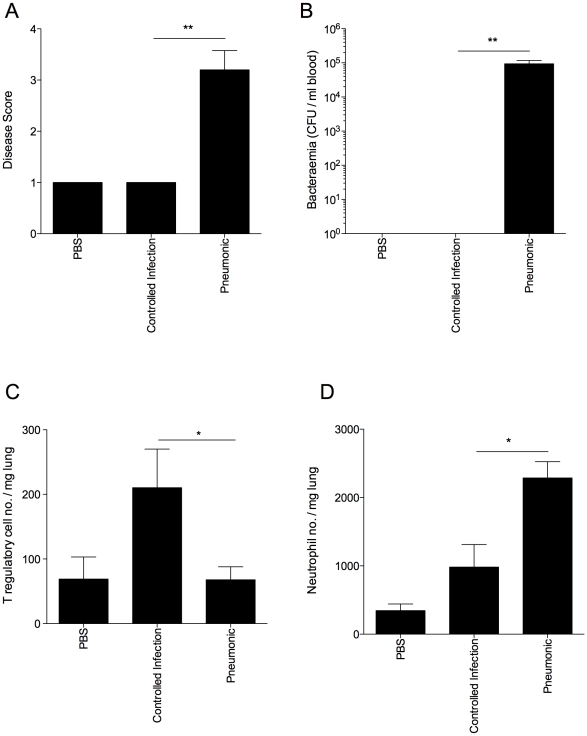
Disease resistance in outbred MF1 mice correlates with strong T regulatory cell responses in the lung. All results are from intranasal-infection of outbred MF1 mice with wild-type *S. pneumoniae* D39. (A) Animal disease score at 48 hours p.i. A score of 1 is a normal mouse. Any infected mouse scoring 1 at 48 hours p.i. was considered to have a controlled infection. (B) Number of bacteria/ml blood at 48 hours p.i. CFU=colony forming units. Number of Foxp3^+^ T regulatory cells (C) and Gr-1^hi^F4/80^lo^ neutrophils (D) per mg lung in PBS-treated or *S. pneumoniae*-infected MF1 mice at 48 hours p.i., as identified in flow cytometry. *'s indicate significant difference, where *=p<0.05 and **=p<0.01. For all graphs data represent mean +/− SEM. Results are representative of 2 independent experiments, each with 3 PBS-treated and >10 infected mice.

To test the hypothesis that strong T regulatory cell responses correlate with resistance to pneumococcal pneumonia in other mouse strains as well, we infected outbred MF1 mice and assessed the progression of disease in relation to T regulatory cell activity (Figure 7). At 48 hours p.i., mice showing visible signs of disease were considered to have invasive pneumonia (Figure 7 A). The remaining mice, which showed no disease signs, were deemed to have “controlled infection" or non-invasive pneumonia. This was confirmed by analysis of bacteraemia at 48 hours p.i., as all mice which showed visible signs of disease had bacteria in their blood compared with no bacteraemia in mice in the non-invasive pneumonia group (Figure 7 B). Strikingly, the resistant phenotype correlated with significantly higher numbers of T regulatory cells in the lungs of mice from the non-invasive pneumonia group as compared to the invasive pneumonia group at 48 hours p.i. (p<0.05) (Figure 7 C). Intriguingly, neutrophil numbers in the lungs of the invasive pneumonia group were significantly higher than in the non-invasive pneumonia group at the same time point (p<0.05) (Figure 7 D), suggesting that neutrophil influx alone is insufficient to mediate resistance to infection in the absence of protective T regulatory cell responses.

## Discussion

Collectively, these data demonstrate an important role for T regulatory cell activity in mediating resistance to pneumococcal pneumonia. Thus, the differing susceptibilities of BALB/c and CBA/Ca mice can be attributed in large part to differences in the co-ordination and regulation of TGF-β signaling pathways in these mice.

Evidence from other diseases suggests that CD4^+^Foxp3^+^ T regulatory cells play prominent roles in immune suppression [36]. Indeed, in some infectious diseases, down-regulation of T regulatory cells by pathogens is a feature of infection that correlates with poor prognosis [37]. It is well known that infection with *S. pneumoniae* results in a rapid and vigorous immune response in the lungs characterized by early infiltration of neutrophils and increased macrophage numbers [38] followed by an influx of lymphocytes [39], but an uncontrolled and sustained influx of pro-inflammatory leukocytes to the lung would be highly detrimental to the host. Excessive inflammatory infiltrate can lead to substantial tissue damage through the actions of degradative enzymes and other soluble mediators released from the infiltrating cells and such tissue damage could compromise the integrity of the epithelial cell barrier of the lung and aid bacterial dissemination to the bloodstream. Therefore, appropriately regulated immune responses in the lung during pneumococcal infection are essential, and T regulatory cells and their products clearly play an important role in this.

It appears that, in the absence of rapid T regulatory cell induction in CBA/Ca mice, bacteria disseminate into the blood stream before a regulated inflammatory environment of infiltrating leukocytes has been established. By contrast, such an environment is generated as early as 12 hours p.i. in BALB/c mice which likely helps to contain the bacteria within the lungs and eventually clear them. T regulatory cells in such an environment might limit the inflammatory reaction in order to prevent tissue damage and preserve epithelial barrier integrity. In CBA/Ca mice, with fewer T regulatory cells, less TGF-β and no immunosuppression, this process is inefficient and may lead to compromise of the lung architecture. This is supported by the substantially increased levels of apoptosis within the lungs of CBA/Ca mice, and increased levels of bacterial seeding into blood.

The correlation between strong T regulatory cell responses and decreased IFN-γ production has been made previously. A recent study outlined a correlation between increased Foxp3^+^ T regulatory cells in Malawian adults (with asymptomatic HIV infection) and an associated skewing of T cell cytokine production away from IFN-γ [40]. In our study, the T cell contribution to IFN-γ production was minimal but we did observe reduced NK cell-derived IFN-γ in BALB/c mice, which have strong T regulatory cell responses.

The finding that pneumococcal pneumonia induces IL-10 production in the lung is a significant one. T regulatory cell-derived IL-10 has a well-documented role in immune homeostasis in the lung during allergy. For example, mice in which IL-10 was specifically ablated in T regulatory cells displayed perivasculitis and mononuclear cell infiltration around the large airways of the lung [41]. The same mice also displayed exacerbated disease signs in an ovalbumin-driven model of allergic airways inflammation. Thus, it appears that T regulatory cell-derived IL-10 plays an important role in the suppression of immunological reactivity in the lung in the context of both allergic inflammation and infection, where it may help limit infection-related tissue damage. In addition to IL-10 production, the T regulatory cells induced in response to pneumococcal infection might mediate immune regulation and the suppression of pathological inflammatory responses through a direct cell-cell contact mechanism. The finding that CTLA-4 is highly expressed on a subset of T regulatory cells in resistant BALB/c, but not susceptible CBA/Ca mice following pneumococcal infection supports this assertion.

Interestingly, our studies in outbred MF1 mice provided complementary evidence for a link between resistance to invasive disease and high lung regulatory T cell numbers but also suggested that uncontrolled lung neutrophil influx in susceptible mice might contribute to lung tissue damage that promotes bacterial dissemination. A similar destructive role for neutrophils has been suggested previously after it was demonstrated that depletion of neutrophils with monoclonal antibodies could prolong survival in mice given a lethal intranasal dose of serotype 8 pneumococci [42]. Furthermore, the detrimental effects of excessive neutrophil recruitment have been documented recently in a study investigating sex-based differences in susceptibility to pneumococcal disease [43]. The greater susceptibility of male mice to invasive pneumococcal disease was attributed in part to the uncontrolled presence of pro-inflammatory cytokines and greater neutrophil infiltration into lungs following respiratory pneumococcal infection as compared to reduced pro-inflammatory cytokines and lower neutrophil infiltration in lungs of female mice. However, these data must be balanced against the proven role for neutrophils in bacterial clearance. It is likely that it is the timing of the neutrophil influx that is crucial [1]–[3], [22]. If recruitment can occur whilst the bacteria are still confined to the lung then disease resolution may occur, but if the influx occurs later, the neutrophils may fail to clear the infection and may aid bacterial dissemination via contribution to lung damage. The earlier influx of neutrophils to the lungs in BALB/c versus CBA/Ca mice certainly correlates with bacterial clearance.

Collectively, these findings dramatically alter our perception of host immune responses to pneumococcal infection, emphasizing the importance of balancing pro-inflammatory responses in the lung with controlled anti-inflammatory and immunomodulatory activity. Furthermore, it is clear that achieving this balance is fundamental to containment and clearance of pneumococcal infection in lungs. The finding that the correlation between strong regulatory cell responses and invasive disease resistance is not restricted to BALB/c and CBA/Ca mice but can also be observed in outbred MF1 mice is an important one, as these genetically variable populations mimic more closely the spectrum of disease resistance phenotypes seen in human populations. In this paper we show how differences in the regulation and balance of host immune responses can dramatically alter the development and outcome of infection by either leading to containment and clearance of the pathogen from lungs or the development of severe invasive pneumonia and lethal septicaemia. It follows that genetic differences in components of immunomodulatory pathways may predispose individuals to the development of septicaemia following acute pneumonia. An improved understanding of the mechanisms operating in the lungs and the role that regulatory cells and cytokines play in these processes may allow us to predict outcomes of infection and tailor treatments accordingly. It will also certainly be of interest to develop novel pneumococcal vaccines that can induce protective T regulatory cell responses.

## Methods and Materials

### Ethics statement

This study was performed in strict accordance with U.K. Home Office guidelines. The protocol was approved by both the U.K. Home Office and the University of Leicester ethics committee. Every effort was made to minimize suffering and in bacterial infection experiments mice were humanely culled if they became lethargic. All animal experiments were carried out at the University of Leicester.

### Mice

Female BALB/cOlaHsd (abbreviated to BALB/c), CBA/CaOlaHsd (CBA/Ca) and MF1 mice were purchased from Harlan Laboratories (Bicester, UK) and were acclimatised for two weeks prior to use. Mice used for infection experiments were between 9 and 14 weeks of age and were age matched for each experiment. The University of Leicester Ethical Committee and the U.K. Home Office approved the experimental protocols.

### Bacteria

*Streptococcus pneumoniae* strain D39 (NCTC 7466) was used for infection. D39 is a mouse virulent serotype 2 strain. Bacteria were identified as pneumococci by the Gram stain, catalase test, α-haemolysis on blood agar and by optochin sensitivity. Presence of capsule was confirmed via quellung reaction. Before use in infection studies, bacteria were passaged through mice to standardize the inoculum and were stored at −80°C. When required, suspensions were thawed at room temperature and the infectious dose prepared as described previously [4].

### Infection of mice

Animals were anaesthetised with a mixture of O_2_ and isofluorane and infected intranasally with 1×10^6^ CFU *S. pneumoniae*, as described previously [4]. Mice were periodically scored for clinical signs of disease and were culled when they became severely lethargic or else at pre-determined times post-infection. Signs of disease were scored based on the scheme of Morton [44]. Mice were culled by cervical dislocation and the lungs were removed and snap frozen in liquid nitrogen or else prepared for flow cytometry by incubation for 30 minutes at 37 degrees with 10 mg/ml collagenase D (Amersham), homogenization and lysis of erythrocytes. Blood samples were taken by cardiac puncture under terminal anaesthesia or from tail-bleeds.

### RNA amplification and labelling for microarray

RNA was pooled for each strain (5 mice per pool). RNA samples were amplified and labelled using the Ambion Amino Allyl MessageAmp aRNA Amplification kit. For each sample 2 µg total RNA was amplified and labelled according to the manufacturer's instructions. Universal Mouse Reference RNA (Stratagene) was used as a reference for gene-profiling.

### Microarray

Samples were hybridized to microarray slide sets consisting of 31,769 oligonucleotides (70mers) from the Array Reagy Oligo set for the Mouse Genome Version 3 (Qiagen). The oligos were distributed over 3 slides and were spotted on codelink slides (Amersham Biosciences) using the Amersham Lucidea spotter. Slides were scanned using the GenePix Pro 4000B scanner (Axon) and Genepix pro 4.1 software. Spots were flagged if the fluorescent signal from a particular spot was no higher than the local background fluorescence or if there was more than a 10% difference in the mean and median intensity of a feature. Signal intensity data were scaled to the reference channel and analysed using the “linear models for microarray data" software Limma in the BioConductor package for R (http://bioconductor.org/biocLite.R).

### Ingenuity Pathway Analysis (IPA)

IPA software (www.ingenuity.com) was used to explore pathways and relationships between genes of interest in a data set.

### qRT-PCR

cDNA was synthesised from RNA using TaqMan Reverse Transcription reagents kit, as per the manufacturer's instructions (Applied Biosystems N808-0234). Real time qRT-PCR was performed using Taqman chemistry. The Taqman assays were pre-made validated FAM-labelled primer-probe sets from Applied Biosystems (for *hprt1*, Mm00446968_m1; for *tgfb1*, Mm03024053_m1). Each sample and assay was performed in triplicate. The PCR reactions were run on an ABI Prism 7500 FAST system (Applied Biosystems) using the standard conditions for Taqman assays. Results were normalised to the house keeping gene *hprt1* and analysed using the 2^−ΔΔCt^ method. For the t=0 samples the sham and infected for each strain were combined and the CBA/Ca samples were compared to the BALB/c samples (the calibrator) to give the fold change (2^−ΔΔCt^) difference. For the t=6 and t=12 samples the infected were compared to the sham (the calibrator) for each sample to calculate the fold change (2^−ΔΔCt^) in response to infection.

### Determination of bacterial numbers in lung and blood

The viable count of bacteria in blood and lungs was determined at the pre-chosen intervals after intranasal infection. Viable counts in lung cell suspension and in blood samples were determined by serial dilution in sterile 1× PBS and plating on blood agar containing 5% (v/v) defibrinated horse blood (Oxoid). Once dry, plates were incubated in 5% (v/v) CO_2_ at 37°C overnight and bacterial colony numbers assessed the following day.

### P17 peptide challenge

500 µg P17 peptide in 100 µl PBS was administered by intraperitoneal (i.p.) injection 1 hour before and 6 hours after intranasal challenge with wild type D39 *S. pneumoniae*. Control animals received i.p. injections of PBS as a control.

### Flow cytometry

Mouse tissue cell suspensions, at 2×10^8^ cells/ml were incubated with purified anti-Fc receptor blocking antibody (anti-CD16/CD32) before addition of the specific antibodies. Cell surface markers were stained using a combination of fluorescein isothiocyanate (FITC)-, phycoerythrin (PE)-, PE-Cy7- and allophycocyanin (APC)-conjugated monoclonal antibodies. Intracellular staining with monoclonal antibodies for Foxp3, Helios, IL-10 and IFN-γ was performed according to the manufacturer's instructions (eBioscience). In each experiment the appropriate isotype control monoclonal antibodies and single conjugate controls (eBioscience) were also included. Samples were analysed using a Becton Dickinson FACScalibur flow cytometer running CellQuest acquisition and analysed using FlowJo software (version 8.8.3, Tree Star).

### T regulatory cell culture

CD4^+^ CD25^−^ T cells were isolated from CBA/Ca spleens by a combination of magnetic depletion and positive selection for CD4, according to manufacturer's instructions (Stemcell Technologies). Cells were cultured at a density of 2×10^6^ cells/ml in 24-well plates for 5 days in X-Vivo15 serum-free medium supplemented with anti-CD28 (2 µg/ml), plate-bound anti-CD3 (10 µg/ml), and TGF-β1 (5 ng/ml) to induce differentiation of Foxp3^+^ CD25^+^ T regulatory cells [35]. CD4^+^ T cells cultured as controls were subjected to the same culture conditions but without TGF-β1.

### Adoptive transfer

100 µl of *in vitro* generated CD4^+^ CD25^+^ Foxp3^+^ T regulatory cells (3×10^6^/ml) in sterile PBS was adoptively transferred into CBA/Ca mice via injection into the tail vein, 2 hours prior to intranasal infection with *S. pneumoniae*. Control CBA/Ca mice received either 100 µl sterile PBS or 100 µl CD4^+^ T cells (3×10^6^/ml) i.v.

### Preparation and analysis of lung tissue section

At necropsy, tissue samples were immersed in 10% v/v formalin saline solution prior to conventional processing and embedding in paraffin wax. Histopathological assessment was performed on tissue sections stained with hematoxylin and eosin (BDH) or Giemsa. Foxp3 staining was performed using antibody clone FJK-16s (eBioscience) and streptavidin horseradish peroxidase chemistry following standard protocols (DAKO). TGFB1 immunostaining was performed with a polyclonal rabbit antibody (Sc-146, Santa Cruz). Counting was performed double-blind using 3 slides per lung per timepoint and 4–5 lungs per timepoint for each strain. Counting was performed on computer on a captured image of the slide.

### Quantification of apoptosis in lung tissues

Rabbit anti-active caspase polyclonal antibody (AF835, R&D systems) was used as a marker of apoptosis. Biotinylated anti-rabbit conjugate (Vector labs) was used as a secondary and this was detected using the Vectastain elite ABC kit (Vector labs). The antibody complex was visualized by the hydrogen peroxide-diaminobenzidene (DAB) reaction, producing a brown end product. Slides were counterstained with hematoxylin prior to examination by light microscopy. The number of brown-staining apoptotic cells were expressed as a percentage of total cells as assessed by number of blue-staining nuclei. Lung tissue was split into alveolar, airway and inflammatory areas and the three regions counted separately. Counting was performed double-blind using 3 slides per lung per timepoint and 4–5 lungs per timepoint for each strain. A minimum of 200 cells were counted per slide and counting continued until either at least 20 positive cells were counted or a maximum of 3000 counted cells was reached. Counting was performed on computer on a captured image of the slide.

### Statistics

Data were analysed using a two-tailed unpaired Student T test or one- or two-way ANOVA, as appropriate. Results with p-values less than 0.05 were considered significant. Statistical analysis was performed with the aid of Graph Pad Prism.

## Supporting Information

Figure S1 **BALB/c mice control** ***S. pneumoniae*** **infection whilst CBA/Ca mice do not.** BALB/c and CBA/Ca mice were intranasally-infected with wild type D39 *S. pneumoniae* and lung (A) and blood (B) bacterial numbers assessed at 0, 12 and 24 hours p.i. Data are representative of >10 independent experiments with at least 5 mice per group. Data represent mean +/− SEM.
(TIF)

Figure S2 **CTLA-4high T regulatory cells increase following** ***S. pneumoniae*** **infection in BALB/c but not CBA/Ca mice.** BALB/c and CBA/Ca mice were intranasally-infected with wild type D39. Data are from a single experiment with 4 mice per group per time point. White bars=BALB/c. Black bars=CBA/Ca. Data represent mean +/− SEM. ***=p<0.001.
(TIF)

Figure S3 **There is no significant difference in IL-17+ cell numbers in BALB/c or CBA/Ca lungs at 24 hours post-infection.** BALB/c and CBA/Ca mice were intranasally-infected with wild-type D39 or PBS as a control. PBS group contains BALB/c and CBA/Ca animals. Data are representative of two independent experiments with >4 mice per group. IL-17+ cell number was assessed by flow cytometry. ns=not significant.
(TIF)

Figure S4 **Neutrophils and macrophages increase significantly in BALB/c but not CBA/Ca lungs following infection.** (A) PMN (Gr-1+, F4/80low) and (B) macrophage (F4/80high) number per mg lung as assessed by flow cytometry. *=p<0.05, **=p<0.01, ***=p<0.001. Data are representative of two independent experiments with >5 mice per group per time point.
(TIF)
