# Supplementary figures and images for: T Regulatory Cells Control Susceptibility to Invasive Pneumococcal Pneumonia in Mice
Source: PLoS Pathog. 2012 Apr 19;8(4):e1002660. doi: 10.1371/journal.ppat.1002660 (PMC3334885; doi:10.1371/journal.ppat.1002660)

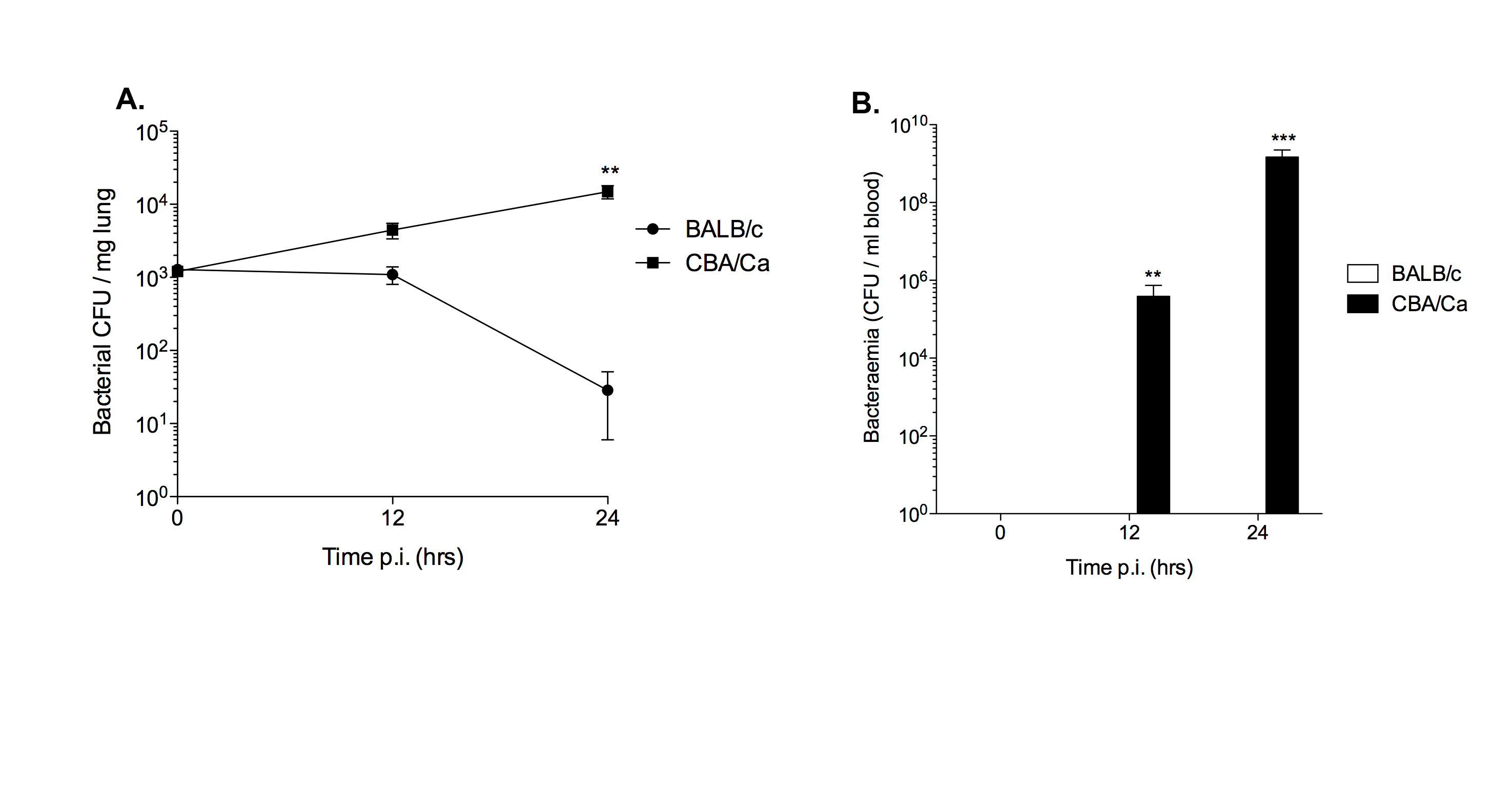

Supplement: Figure S1 — BALB/c mice control S. pneumoniae infection whilst CBA/Ca mice do not. BALB/c and CBA/Ca mice were intranasally-infected with wild type D39 S. pneumoniae and lung (A) and blood (B) bacterial numbers assessed at 0, 12 and 24 hours p.i. Data are representative of >10 independent experiments with at least 5 mice per group. Data represent mean +/− SEM. (TIF) [file ppat.1002660.s001.tif]

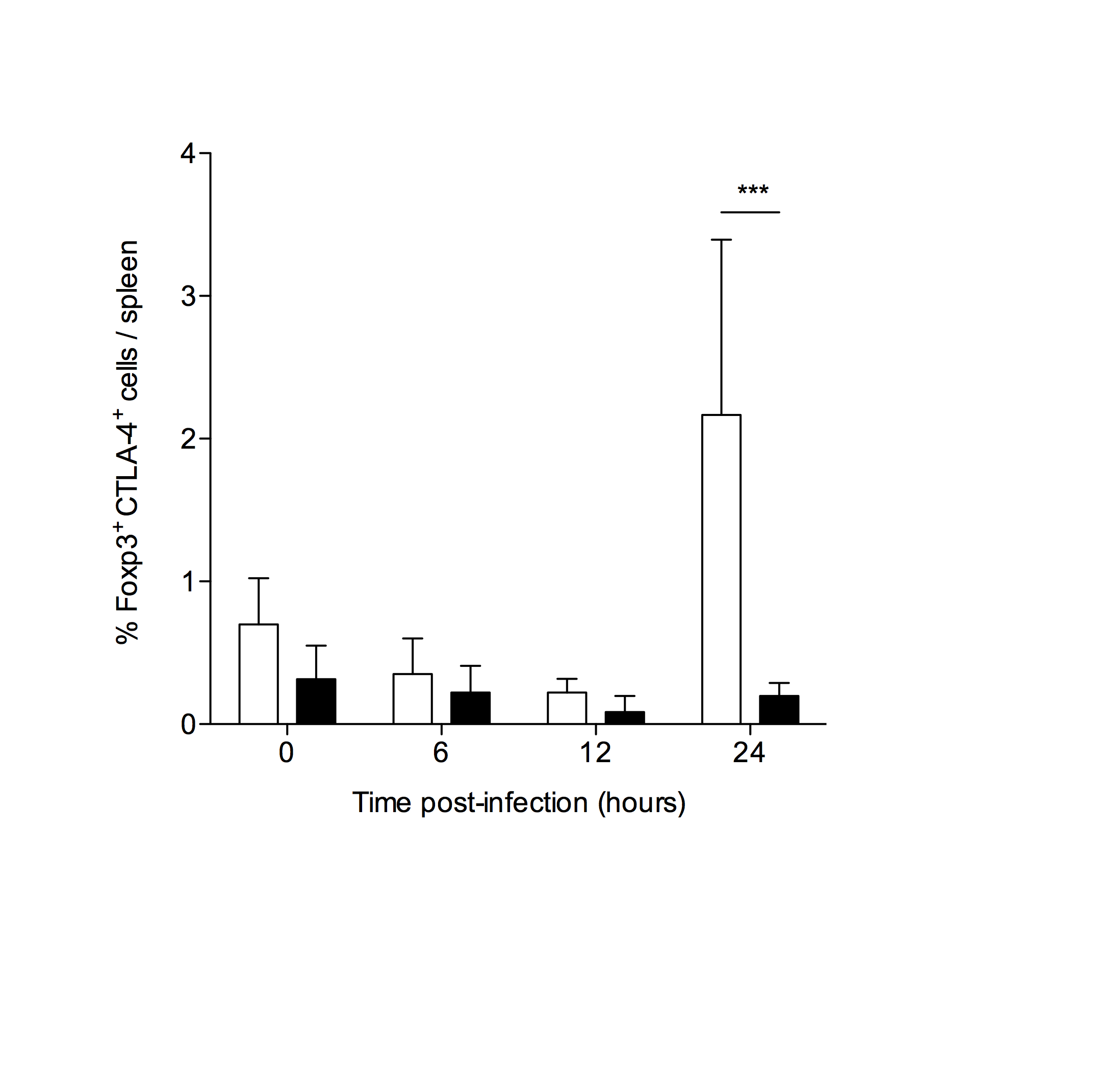

Supplement: Figure S2 — CTLA-4high T regulatory cells increase following S. pneumoniae infection in BALB/c but not CBA/Ca mice. BALB/c and CBA/Ca mice were intranasally-infected with wild type D39. Data are from a single experiment with 4 mice per group per time point. White bars=BALB/c. Black bars=CBA/Ca. Data represent mean +/− SEM. ***=p<0.001. (TIF) [file ppat.1002660.s002.tif]

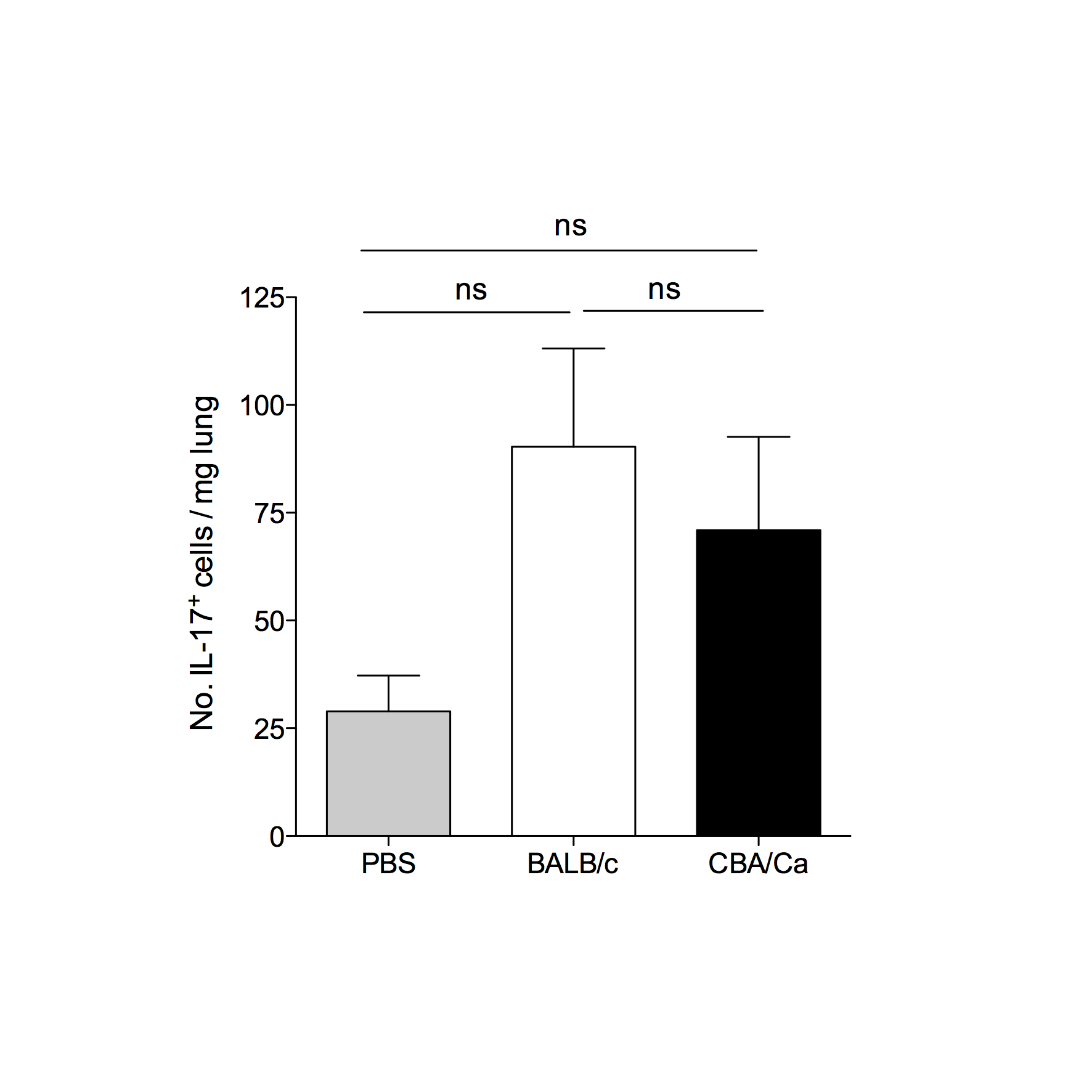

Supplement: Figure S3 — There is no significant difference in IL-17+ cell numbers in BALB/c or CBA/Ca lungs at 24 hours post-infection. BALB/c and CBA/Ca mice were intranasally-infected with wild-type D39 or PBS as a control. PBS group contains BALB/c and CBA/Ca animals. Data are representative of two independent experiments with >4 mice per group. IL-17+ cell number was assessed by flow cytometry. ns=not significant. (TIF) [file ppat.1002660.s003.tif]

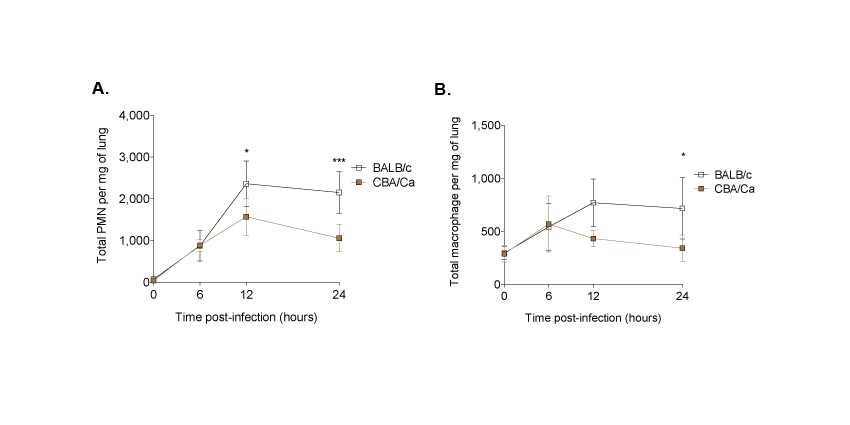

Supplement: Figure S4 — Neutrophils and macrophages increase significantly in BALB/c but not CBA/Ca lungs following infection. (A) PMN (Gr-1+, F4/80low) and (B) macrophage (F4/80high) number per mg lung as assessed by flow cytometry. *=p<0.05, **=p<0.01, ***=p<0.001. Data are representative of two independent experiments with >5 mice per group per time point. (TIF) [file ppat.1002660.s004.tif]
